# Mobile Phonocardiogram Diagnosis in Newborns Using Support Vector Machine

**DOI:** 10.3390/healthcare5010016

**Published:** 2017-03-18

**Authors:** Amir Mohammad Amiri, Mohammadreza Abtahi, Nick Constant, Kunal Mankodiya

**Affiliations:** 1 Department of Physical Therapy, College of Public Health, Temple University, Philadelphia, PA 19140, USA; 2 Department of Electrical, Computer, and Biomedical Engineering, University of Rhode Island, Kingston, RI 02881, USA; mabtahi@ele.uri.edu (M.A.); kabuki4774@gmail.com (N.C.); kunalm@uri.edu (K.M.)

**Keywords:** m-health, phonocardiogram, SVM

## Abstract

Phonocardiogram (PCG) monitoring on newborns is one of the most important and challenging tasks in the heart assessment in the early ages of life. In this paper, we present a novel approach for cardiac monitoring applied in PCG data. This basic system coupled with denoising, segmentation, cardiac cycle selection and classification of heart sound can be used widely for a large number of the data. This paper describes the problems and additional advantages of the PCG method including the possibility of recording heart sound at home, removing unwanted noises and data reduction on a mobile device, and an intelligent system to diagnose heart diseases on the cloud server. A wide range of physiological features from various analysis domains, including modeling, time/frequency domain analysis, an algorithm, etc., is proposed in order to extract features which will be considered as inputs for the classifier. In order to record the PCG data set from multiple subjects over one year, an electronic stethoscope was used for collecting data that was connected to a mobile device. In this study, we used different types of classifiers in order to distinguish between healthy and pathological heart sounds, and a comparison on the performances revealed that support vector machine (SVM) provides 92.2% accuracy and AUC = 0.98 in a time of 1.14 seconds for training, on a dataset of 116 samples.

## 1. Introduction

The leading cause of death in the United States in 2011 was heart diseases, killing nearly 787,000 people [1]. In a report in 2014 by American Heart Association (AHA), 17.3 million deaths caused by cardiovascular disease per year led heart diseases to be the No. 1 global cause of death for more than 190 countries, and this is expected to grow to more than 23.6 million by 2030 [2].

Although heart diseases are counted as a major cause of death, diagnosing them at an early stage of life can dramatically reduce most risk factors. Some heart diseases can cause life-threatening symptoms and require intervention within the first days or weeks of life as even critical congenital diseases are often treatable upon early detection.

Despite remarkable advances in imaging technologies for heart diagnosis, clinical evaluation of cardiac defects by auscultation is still a main diagnostic method for discovering heart disease. In experienced hands, this method is effective, reliable, and cheap. Phonocardiography (PCG) contains information which is widely used by physicians to evaluate cardiac functions in patients and detect the presence of abnormalities [3].

A newborn’s heart still develops within the first days or weeks of life; therefore, there are extra sounds called Innocent murmurs. Also, the existence of an extra sound which is a heart problem that a baby is born with, is called a pathological murmur or congenital heart defect [4]. Heart murmurs are very common and occur in up to 80% of children at some time or another. It is very important to distinguish between innocent and pathological murmurs.

The most common cause of innocent murmurs in newborns is when a specific condition called Patent Ductus Arteriosus (PDA) occurs, which is often detected shortly after birth and most commonly in premature newborns. PDA is a potentially serious condition in which blood circulates abnormally throughout the ductus arteriosus. In general, the only symptom of PDA is a heart murmur, which lasts until the ductus closes on its own, which for healthy (innocent) newborns, usually closes within the first hours of life. Sometimes, especially in premature newborns, it does not close on its own, or it may be large and permit too much blood to pass through the lungs, which can place extra strain on the heart, forcing it to work harder and causing a rise in blood pressure in the arteries of the lungs. In this case, a medication or, rarely, surgery may be needed to help close the PDA [5].

**Figure 1:**
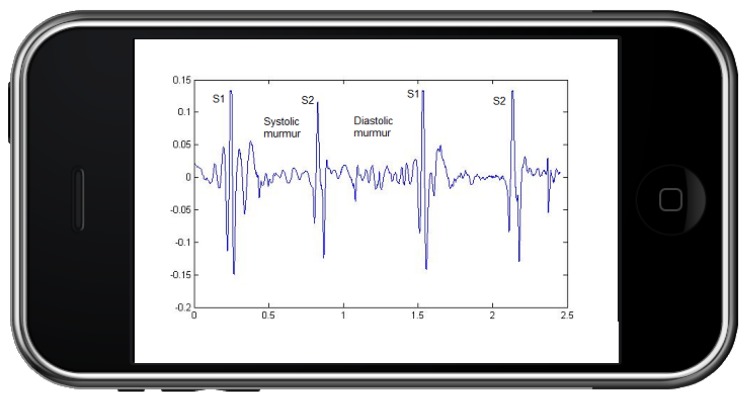
An illustration of the Phonocardiogram with its components.

Phonocardiography includes two major sounds: The first heart sound is caused by the closing of the mitral and tricuspid valves, and the second heart sound is created by the closing of the aortic and pulmonic valves. The interval between two sounds is called a murmur. There are two murmurs: Systolic murmurs occur between S1 and S2 (first and second heart sounds) and, therefore, are associated with mechanical systolic and ventricular ejection. Diastolic murmurs occur after S2 and before S1; they are therefore associated with ventricular relaxation and filling. Diastolic murmurs include aortic and pulmonic regurgitation (early diastolic), and mitral or tricuspid stenosis (mid-late diastolic). Tricuspid stenosis is very rare and is discussed further in the valvular heart disease section (see Figure 1) [6].

Diagnose of the heart murmur in the healthy/unhealthy newborn is very common and takes several days or weeks to disappear. Usually, the exact cause of the problems are unknown, but there is often a chance that it is due to the complex development of the newborns heart. For example, PDA is the most common heart murmur in newborns; it is known as a pathological murmur if it does not disappear shorty after birth. Therefore, it is very important to monitor newborns’ heart in order to reduce the serious risk of heart problems in the future.

Real time monitoring of PCG is an important diagnostic tool to measure and analyze the functional status of the heart. As the volume of the data increases, the complexity and the relationships underneath the data also increases. In an early stage of data collection and transmission in information systems, the focus is on finding the best feature values to represent each observation.

It is a challenging venture to remote monitor newborns for distinguishing pathological murmurs from innocent ones. In this study, we propose a method to automatically classify PCG data, separating innocent from physiological murmurs. A novel technique is also implemented for PCG segmentation. Feature extraction has been performed in time and frequency domain which was very effective in improving experimental results. The method utilizes different types of classifiers and shows that a support vector machine is a suitable choice to identify pathological murmurs.

## 2. Related Works

The recent study by J. Herzig [7] presented a type of cardiac monitoring based on heart sound analysis. Specifically, the study uses two morphological features and their associations with physiological changes from the baseline state. The framework is demonstrated on recordings during laparoscopic surgeries of 15 patients. The paper demonstrates that the proposed features change during cardiac stress, and the change is more significant for patients with cardiac problems. Furthermore, they showed that other well-known ECG morphology features are less sensitive in this specific cardiac stress experiment.

Another paper by S. Barma [8] reports a feature generation method for automatic heart sound monitoring system based on the nonlinear signal decomposition and the instantaneous characteristics of the decomposed components. In this work, the heart sounds (normal and abnormal) are decomposed by complementary ensemble empirical mode decomposition. The experiment is performed on total 23 different classes of heart sounds including the normal and abnormal cases, collected from the Michigan Heart Sound and Murmur Database. The results indicate that the proposed method can achieve a recognition rate of 98%.

A publication by Kovcs, Ferenc et al. [9] presents a complex heuristic method for the evaluation of fetal heart sounds, applying simultaneously several algorithms, where the autocorrelation technique has been completed with the wavelet transform and the matching pursuit methods. In this way, a more reliable heart rate variability can be achieved and further parameters of the cardiac operation can be assessed in addition to the conventional cardiotocographic examination. This also comprises those parameters, which can be investigated only with long-term or continuous monitoring, and those, which rely on a very accurate estimation of the heart rate variability.

Maglogiannis [10] et al., proposed a diagnosis system using support vector machine to identify heart valve diseases. They applied the system on a dataset of 198 heart sound signals from healthy medical cases and cases with four most usual heart valve diseases: aortic stenosis (AS), aortic regurgitation (AR), mitral stenosis (MS) and mitral regurgitation (MR). The system categorized the heart sounds as normal or disease-related and then the corresponding murmurs in the unhealthy cases were classified as systolic or diastolic. This study also applied some different classifiers to the same dataset for comparison (i.e., back-propagation neural networks, k-nearest-neighbour and naïve Bayes classifiers) and showed that the SVM classifier worked better for the same diagnostic problems.

Recently, another report by Shuang Leng et al. [11] provided a study of the electronic stethoscope technology, and the methodology for diagnosis of cardiac disorders based on computer-aided auscultation. The paper is based on a comprehensive literature review of articles, market products, and smartphone stethoscope apps. It covers some key component of the computer-aided system with electronic stethoscope, from sensor design, front-end circuitry, denoising algorithm, heart sound segmentation, to the final machine learning techniques. The authors tried to provide an informative and illustrative presentation of the electronic stethoscope, which is valuable and beneficial to academics, researchers and engineers in the technical field, as well as to medical professionals to facilitate its use clinically. The paper provided the technological and medical basis for the development and commercialization of a real time integrated heart sound detection, acquisition and quantification system.

This study presents novel methods in segmentation (S1 and S2 detection), cardiac cycle selection for PCG signal reduction and a classification for distinguishing heart murmurs which can be used widely in data analysis applications.

## 3. Methods

**Figure 2:**
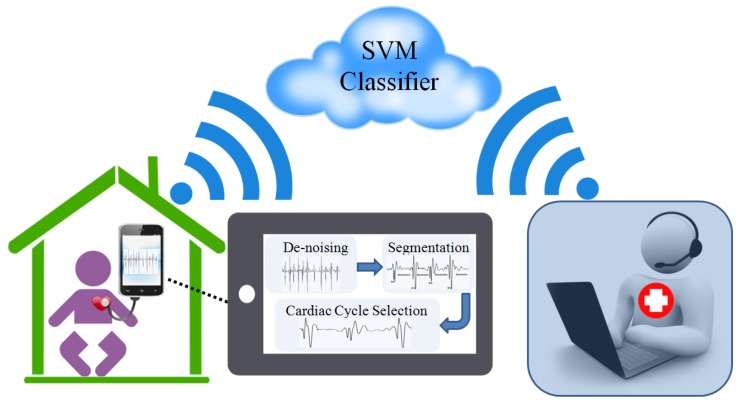
Schematic of telemedication frame work used in this study.

This phase involves pre-processing and features extraction of the signal that is certain characteristic properties of heart sound that are unique to the signal and are thus suitable for classification purposes. Figure 2 shows stages used in the proposed system.

### 3.1. Data Acquisition

PCG data has been recorded by an electronic stethoscope which was connected to a mobile device. We recorded the PCG data from 116 newborns who were 1–20 days old at Imam Ghaem Hospital, Mashhad, Iran. All newborns were visited by a cardiologist who used echocardiography to label the PCG data with information on murmurs. The label is used for specifying whether that murmur is innocent or pathological. We received the consent of all parents.

### 3.2. De-Noising

The goal of de-noising and filtering the PCG data is to remove or reduce the undesired noise. PCG data coupled with noise could be expected when there is noise in the environment of recording the PCG data which is usually recorded in sampling frequencies of more than 8 kHz. Therefore, filtering the data plays an important role in order to give a wide berth to unpredictable affects which can definitely affect the later processing stages performance. In order to record the heart sound, an electronic stethoscope has been used which records the data at 44 kHz. A very useful tool for visualizing the spectrum of the frequencies of a signal varying with time is spectrogram. The spectrogram shows the changes in the energy of the signal over the time and frequency components. The spectrogram was applied on a 12 s window in the PCG signal, and it showed that there are relevant changes in the range of 100 Hz. The system uses a 3rd order band-pass Butterworth filter with cut-off frequencies of 20 Hz and 100 Hz in order to get the main spectrum components of first and second heart sound (S1 and S2, respectively).

### 3.3. Segmentation

After de-noising, the next step is to identify the S1 and S2 components of the signal with the timing intervals between them. The following framework is developed in order to make segmentations of the PCG signal into four major areas of S1; S2; systolic and diastolic. The detection of the S1 and S2 could be implemented manually, but we have conceived an algorithm to identify them based on the time interval between high amplitude components. Therefore, we have used the Gabor Wavelet for peak detection which can formally be described as below:

(1) $$\Psi \left(t\right)=C\cdot e^{-jwt}\cdot e^{-t^{2}}$$

where $e^{-jwt}\cdot e^{-t^{2}}$ is the complex Gaussian function and *C* is a normalizing constant.

**Figure 3:**
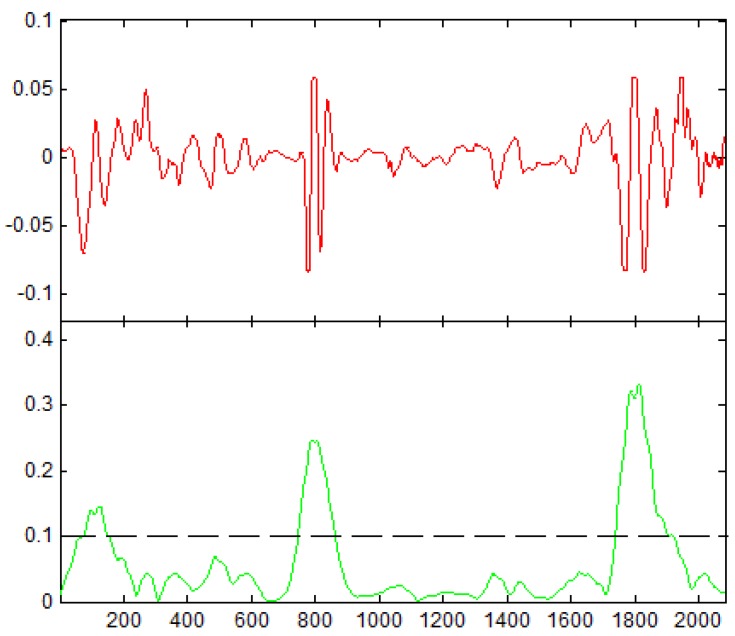
Peak detection using Complex Gaussian Wavelet (Red: raw signal, Green: signal after using Gabor Wavelet).

In order to distinguish the peaks, a threshold set to 0.1 for wavelet scale coefficients have been used (see Figure 3) along with zero-crossing to find the spots where peaks occur.

Also, the number of zero crossings in each segment, represents the dominant component of a signal segment. The algorithm calculates the size of the intervals where the function has the value of zero. It is also important to recall that the smaller and bigger time intervals are related to the systolic (S1 – S2) and diastolic (S2 – S1) murmurs occurrence respectively.

### 3.4. Cardiac Cycle Selection

After detecting cardiac cycles, it is important to identify which cycle shows more signs of heart disease—as detecting the most informative cycle can be very useful for cardiac function assessment. For each patient, recorded data include several cardiac cycles on a time span of few seconds. Despite the fact that filtering has been implemented to remove noise, the residual noise may be part of the PCG signal—such as respiratory sound, artifact noise or newborn voices. Dynamic time warping (DTW) has been used to calculate the similarity among cycles with a pattern cycle which the outcome leads to select a cycle with minimum noise and most properties of the whole signal.

DTW is an algorithm that finds similarity in two sets of time series data using, as a base, a Euclidean distance metric. Its use can be found all around in gait detection, word recognition and in PCG data recognition. The Euclidean algorithm will measure the distance between two points, *x* and *y*, across the sets of time series data. This distance, *p*, is usually denoted by d(x,y) where:

(2) $$d\left(x,y\right)=\sqrt{{\left(x_{1}-y_{1}\right)}^{2}+\cdots +{\left(x_{n}-y_{n}\right)}^{2}}$$

Measuring the distance works well in many cases, but for some special cases, it can come up short. Suppose you have two signals which look similar but are phase shifted. According to the Euclidean distance metric, the distance between the two signals would be large thus meaning there is a little similarity. However, these signals are in fact quite similar only differing by a phase shift. This type of issue can occur when looking at PCG signals, such as when the heart beat increases or decreases in frequency. The difference between simply measuring just the distance metric and DTW is that DTW will utilize dynamic programming to better tackle these special cases.

Let’s denote the two-time series sets as *A* and *B* of lengths m and n respectively. The process starts by creating a *m*-by-*n* adjacency matrix and finds the optimal path from (m,1) to (1,n). This optimal path is found using dynamic programming which adds the distance of the current cell to the minimum distance found in the three adjacent cells. There are methods to speed this process up by creating bounds such that the optimal path is forced into a bounded region along the diagonal. Two popular bounds are the Sakoe-Chiba Band [12] and the Itakura Parallelogram [13].

**Figure 4:**
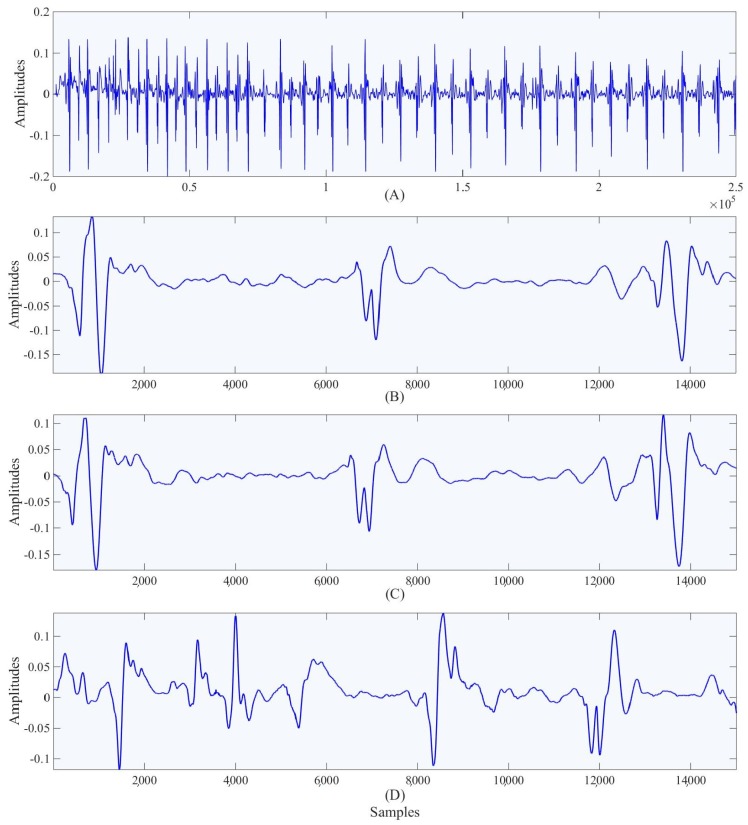
Illustration of Phonocardiography (PCG) signal. Panel (**A**) shows the de-noised data, panel (**B**) shows the selected window of the data, panel (**C**) shows the best window with the distance less than 0.005 from the selected window and panel (**D**) shows a worse window which has distance more than 0.005 from the selected window.

Using DTW, we can determine which segments of the PCG do not match the previously chosen segment. From these poorly matched segments, a second highly correlated segment can be found. After several iterations, we will be left with the fewest number of segments which together show all of the useful information contained within the denoised PCG. The data which then needs to be sent over to the physician can be as small as a single segment of data using these methods. The segments to be sent over are required to have a distance less than 0.005 from the segments of the signal which it is meant to describe. In the Figure 4 shown we were able to reduce the full set of PCG data down to just 2 segments with distances less than 0.005 from the original set. This data reduction can reduce the amount of time the device spends transmitting the data, and it helps the physician by automating the process of looking for sections of importance in the signal.

### 3.5. Feature Extraction

The goal of this phase is to extract features from the PCG signals that are able to represent properties deemed useful for the diagnosis. Different signal processing tools are used on a cloud for detecting heart murmurs.

As for feature extraction, several time-frequency domain’s features have been taken into account, including Maximum Value Amplitude, Sum of Positive Area, Variance, Shannon energy, Bispectrum and Wigner Bispectrum, which have proved to be very effective for improving the performance of classification [14,15].

## 4. Classification

**Figure 5:**
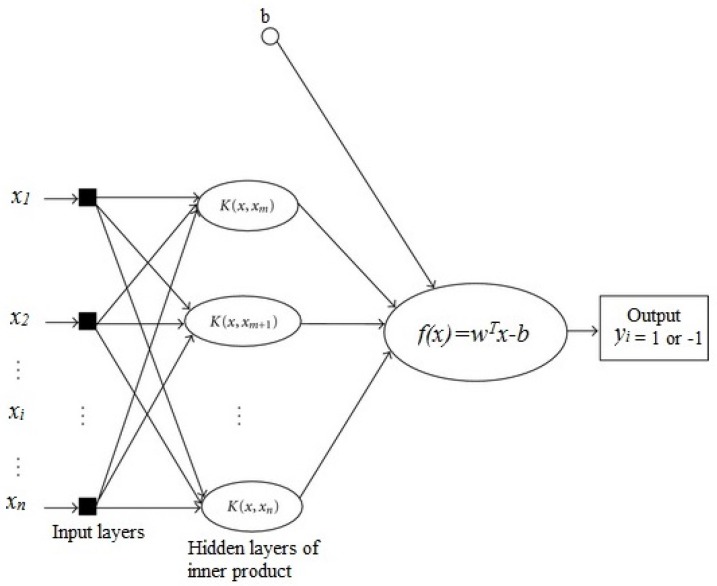
Predicting $y_{i}$ support vector machine architecture.

The Support Vector Machine (SVM), also known as support vector network [16], is a supervised learning method in machine learning and there has been a drastic development in the strategies of the algorithms and its applications in recent years [17]. SVM is mostly used for classification by analyzing data and recognizing patterns with some associated algorithms. Going into more detail, the SVM algorithm makes a model that recognizes the patterns in the given training data set of examples such that each of them belongs to one of two different categories, and then predicts the new examples or features of the data set into one of the two categories. In other words, SVM is a discriminative classifier and the algorithm categorizes new examples on an optimal separating hyperplane or decision boundary (see Figure 5).

There are many different methods of SVM which we are not going into here. If the training data set is linearly separable, then we can use linear SVM by selecting two boundaries that separate the samples. In general, any decision boundary can be described as a set of points of *x* which could satisfy the equation $w^{T}x-b=0$. In this equation, *w* is the orthogonal vector from the origin to the decision boundary and the scalar offset of the decision boundary from the origin can be determined by *b*. Assuming that the features or samples vector are denoted by $x_{i}$ and the categories or the target is denoted by $y_{i}$, which in this study we assigned $y_{i}=+1$ and −1 for healthy and pathological cases, respectively. Based on the values of the $y_{i}$, our decision boundaries would be:

(3) $$w^{T}x-b=1\;\mathrm{and}\;w^{T}x-b=-1$$

It is clear that the distance between these two boundaries, also known as the margin, is 2/|w|. Since one of the goals of the system is to maximize the margin, it would be obvious that it needs *w* to be minimized. Also, we need to prevent the case where the points lie in the margin, so our boundaries constraints would be:

(4) $$w^{T}x-b\geq 1\;\mathrm{and}\;w^{T}x-b\leq -1$$

Multiplying $y_{i}$ and generalizing the equations leads to: $yi(w^{T}x_{i}-b)\geq 1$ for all *i*.

Therefore, the primary form of the linear SVM algorithm would be: $Min(|w{|}^{2}/2)$ with the constraint of $y_{i}\left(w^{T}x_{i}-b\right)\geq 1$ for all *i* [18].

## 5. Experiments and Results

In this study, an SVM was developed in order to diagnose two different PCG signals. A total of 116 PCGs (normal and pathological) were studied. In this section, we present the results of the above proposed Linear SVM technique which include accuracy, Receiver Operating Characteristic (ROC) and training time. K-fold cross validation (K = 8) has been used as a training and test strategy which is based on holdout method. The data set divides into *k* subsets. Each time, one of the *k* subsets is used as the test set and the other k−1 subsets are put together to form a training set.

Classification results of Linear SVM are shown in Table 1 in the form of a confusion matrix, together with the percentage of classification accuracy. It can be seen that out of 58 normal signals, 56 were correctly classified as normal, and 2 were misclassified as pathological. As for 58 pathological signals, 51 were correctly classified as pathological and 7 went through misclassification. A detailed analysis of the misclassified example showed that it was in fact very difficult to classify, even by human experts.

The results verify the validity and performance of the proposed algorithm with 92.2% accuracy. Table 2 shows the results in comparison with other classifiers regarding accuracy, the area under ROC curve and training time. K-fold cross-validation has been used for all classifiers with k=8.

**Figure 6:**
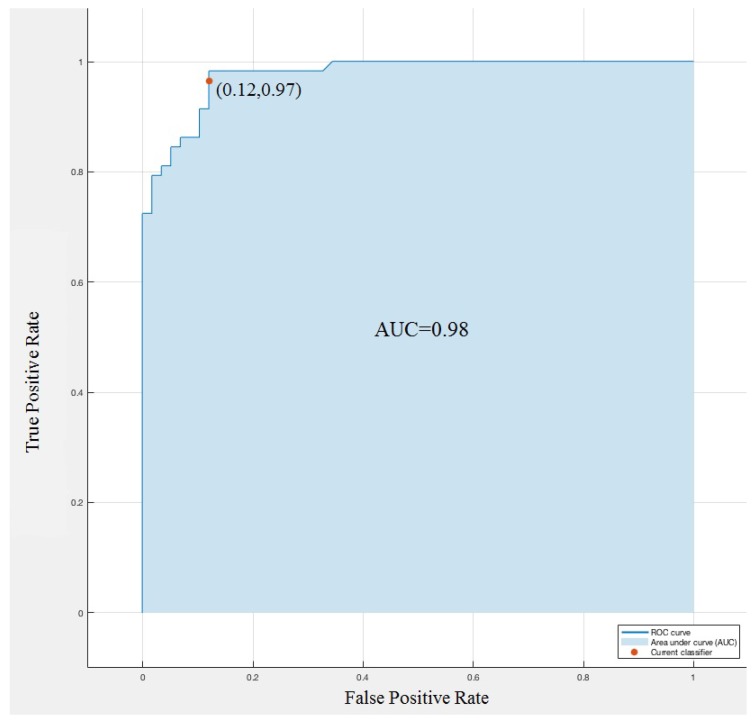
ROC curve of innocent and pathological murmurs classified.

In a Receiver Operating Characteristic (ROC) curve for a binary classification problem, the true positive rate (Sensitivity) is reported as a function of the false positive rate (100-Specificity) for different cut-off points. The ROC curve reported in Figure 6 is targeted for classifying the innocent and pathological murmurs. A predictive model with perfect performance has an area under curve (AUC) equal to 1. We obtained, on average, an accuracy of AUC = 0.98, the ROC curve highlights the excellent performance of Linear SVM to identify heart murmurs.

## 6. Conclusions

In the previous sections, novel methodologies for automated segmentation and cardiac cycle selection on mobile devices have been performed. The proposed system initially includes the three-step diagnosis phase mentioned before following a linear model of support vector machine classifying working on cloud computing. The linear SVM showed promising results with an accuracy of 92% prediction and an area under the ROC curve of 0.98. Other classifying methods were applied on the dataset and none of them could beat the linear SVM model.

We deem that the high performance obtained with screening results is due to a perfect matching among the techniques adopted at various stages of the auto-segmentation, cardiac cycle selection, feature extraction and classification. This paper shows novelties in the methods of data reduction in the PCG data analysis application and the SVM classifier in the cloud computing allows the physician to reach the most accurate data along with the benefits of the reduced data size and selected features.

The proposed technology is intended for high-volume screening of newborns suspected of having a pathological murmur. The software system proposed in this work can be considered as a portable and diagnostic tool which is able to support patients and physicians in their health care task.

## Figures and Tables

**Table 1:** Classification result of heart disease in newborns.

Actual Group	Normal	Pathological	Percent Correct
**Normal**	56 (97%)	2 (3%)	97%
**Pathological**	7 (12%)	51 (88%)	88%
**Average/Overall**	116		92.2%

**Table 2:** Comparison of Accuracy, Area Under Curve and Training Time for different classifiers.

Model	Accuracy	AUC	Training Time
Complex Tree	88.8%	0.90	2.88 s
Simple Tree	86.2%	0.89	0.56 s
Linear Discriminant	73.3%	0.84	1.14 s
Quadratic Discriminant	77.6%	0.85	0.74 s
Logistic Regression	86.2%	0.89	2.72 s
**Linear SVM**	**92.2%**	**0.98**	**1.41 s**
Boosted Trees	78.4%	0.78	4.69 s
Bagged Trees	87.9%	0.96	4.32 s
Subspace Discriminant	85.3%	0.96	4.66 s
Subspace Trees	77.6%	0.86	4.96 s
